# Detection of four rare thalassemia variants using Single-molecule realtime sequencing

**DOI:** 10.3389/fgene.2022.974999

**Published:** 2022-09-02

**Authors:** Shiqiang Luo, Xingyuan Chen, Dingyuan Zeng, Ning Tang, Dejian Yuan, Bailing Liu, Lizhu Chen, Qingyan Zhong, Jiaqi Li, Yinyin Liu, Jianping Chen, Xiaoyuan Wang, Tizhen Yan

**Affiliations:** ^1^ Liuzhou Key Laboratory of Birth Defects Prevention and Control, Department of Medical Genetics, Liuzhou Maternity and Child Healthcare Hospital, Liuzhou, China; ^2^ Liuzhou Key Laboratory of Reproductive Medicine, Liuzhou, China; ^3^ Department of Laboratory Medicine, The People’s Hospital of Guangxi Zhuang Autonomous Region, Nanning, China; ^4^ Guangxi Academy of Medical Sciences, Nanning, China; ^5^ Guangxi Health Commission Key Laboratory of Birth Cohort Study in Pregnant Women of Advanced Age, Liuzhou, China; ^6^ Berry Genomics Corporation, Beijing, China; ^7^ Liuzhou Maternity and Child Healthcare Hospital, Liuzhou, China

**Keywords:** single molecule real-time technology, carrier screening, αthalassemia, *ß*-thalassemia, rarevariant

## Abstract

Conventional methods for the diagnosis of thalassemia include gap polymerase chain reaction (Gap-PCR), reverse membrane hybridization (RDB), multiplex ligation-dependent probe amplification (MLPA) and Sanger sequencing. In this study, we used single molecule real-time technology (SMRT) sequencing and discovered four rare variants that have not been identified by conventional diagnostic methods for thalassemia. We also performed genotype and phenotype analyses on family members of thalassemia patients. The SMRT technology detected five cases in which the proband had abnormal results by conventional diagnostic methods or inconsistencies between the genotype and phenotype. The variants included two cases of an α-globin gene cluster 27,311 bp deletion, --^27.3^/αα (hg38 chr16:158664-185974), one case of an HS-40 region 16,079 bp deletion (hg38 chr16:100600-116678), one case of a rearrangement of -α^3.7^α1α2 on one allele and one case of a *ß*-globin gene cluster *HBG1-HBG2* 4,924 bp deletion (hg38 chr11:5249345-5254268). This study clarified the hematological phenotypes of four rare variants and indicated the application value of SMRT in the diagnosis of rare α-globin and *ß*-globin gene cluster deletions, gene recombination and deletion breakpoints. The SMRT method is a comprehensive one-step technology for the genetic diagnosis of thalassemia and is particularly suitable for the diagnosis of thalassemia with rare deletions or genetic recombination.

## Introduction

Thalassemia is a hereditary blood disease that seriously threatens human health, causing death and disability. Many countries around the world are concerned about this disease that is identified as a birth defect (Bajwa and Basit, 2021; Li et al., 2014). The WHO has estimated that thalassemia gene carriers account for approximately 1.67% of the total world population. Thalassemia is one of the most common genetic diseases in southern China and the high incidence provinces of Guangdong, Guangxi, Yunnan and Hainan have a thalassemia gene carrier rate of between 11% and 25% (Xiong et al., 2010; Zhang et al., 2012; Yao et al., 2014). Thalassemia is a hemoglobinopathy that disrupts the balance of α- and *ß*-globin chains due to mutations, deletions, duplications and gene rearrangements in the α- and *ß*-globin gene clusters. The global carrier rate of thalassemia is approximately 1.67%. The phenotype of individuals with thalassemia varies widely, ranging from asymptomatic carriers, mild anemia, moderate anemia, severe anemia, which requires prolonged blood transfusions to maintain life, more severe cases and even pre or postnatal death (Modell and Darlison, 2008). The severity of the disease is often closely related to the number of globin genes that are nonfunctional. In addition, duplication of the α-globin gene cluster, which exacerbates the α/β imbalance, can aggravate the clinical manifestations of heterozygous *ß*-thalassemia. At present, the important thalassemia control measures are carrier screening and prenatal diagnosis of high-risk couples (Goonasekera et al., 2018). The range of α-globin gene deletions is heterogeneous, ranging from deletions of several thousand base pairs, which only involve the vicinity of two α-genes, to those that involve more than two α-genes. In addition to cases caused by deletion in the α-globin gene, a small number of α-thalassemia cases are caused by point mutations. To date, more than 120 α-globin gene mutations have been identified in the Chinese population (data from the Human Variome Project China, http://www.genomed.zju.edu.cn/LOVD 3/genes). Six mutations (--^SEA^/, -α^3.7^/, -α^4.2^/, *HBA2*:c.369C > G, *HBA2*:c.427T > C and *HBA2*:c.377T > C) account for more than 98% of all α-gene mutations (Harewood and Azevedo, 2021). Therefore, the existing clinical diagnosis routine is mainly aimed at identifying these mutations. *ß*-thalassemia is mainly caused by mutation of the *ß*-globin gene. Most of these mutations are point mutations or small fragment deletions, with a few large fragment deletions (Mettananda and Higgs, 2018). To date, 129 *ß*-globin point mutations and 16 *ß*-thalassemia deletions have been found in the Chinese population (data from HVP China; http://www.genomed.zju.edu.cn/LOVD3/genes) and named in accordance with the Human Genome Variation Society rules (den Dunnen et al., 2016). Eight of these mutations (*HBB:c.124_127delTTCT, HBB:c.52A > T, HBB:c.316-197C > T, HBB:c.-78A > G, HBB:c.216_217insA, HBB:c.79G > A, HBB:c.92+lG > T* and *HBB:c.-79A > G*) account for more than 95% of the *ß*-thalassemia mutations in the Chinese population (Needs et al., 2021).

In recent years, detection methods for α- and *ß*-globin deletions have included multiplex amplifiable probe hybridization (MAPH) (Kousoulidou et al., 2008), MLPA (Shen and Wu, 2009), quantitative multiplex PCR of short fluorescent fragments (QMPSF) (Feuk et al., 2006), real-time quantitative PCR (RT-PCR) (Weaver et al., 2010) and PCR melting curve analysis (PMCA) (Xiong et al., 2011). Each of these methods has advantages and disadvantages for screening and diagnostic purposes. The molecular diagnostic technologies used for the clinical detection of α- and *ß*-globin point mutations include Gap-PCR and RDB. These methods are mainly used to detect four common deletions of α-thalassemia (--^SEA^, -α^3.7^, -α^4.2^, -^THAI^), three non-deletion types of α-thalassemia (α^WS^α, α^CS^α and α^QS^α) and 17 common *ß*-thalassemia mutations in the Chinese population. The MLPA technology is a highly efficient and specific method for screening α- and *ß*-globin copy number variations and can simultaneously detect changes in the copy number of more than 40 target sequences in one reaction. It has been popularized and applied as a routine technology in many large hospitals in economically developed areas of China (Liu et al., 2008). Research teams have also used array comparative genomic hybridization (aCGH) to detect α-thalassemia copy number variation (Phylipsen et al., 2012). High-throughput sequencing technology (NGS) has been used for genetic screening of thalassemia and can effectively detect the genotype, in addition to finding new genetic mutations. This method has the advantages of simple collection of specimens and high accuracy of results (Achour et al., 2021). However, due to the expensive equipment and consumables required for NGS technology, its clinical application has been limited.

In recent years, third-generation sequencing technology has been applied to the detection of the thalassemia gene. This technology is also called SMRT. The SMRT method performs separate sequencing of each DNA molecule and PCR amplification is not required. The technology is able to produce ultra-long read lengths (between 30 and 100 kb) and high accuracy (QV30 > 99.8%), characterized by GC-free preference and single-molecule resolution (Wenger et al., 2019). Using SMRT, one-time full coverage of all mutation types of α-thalassemia and *ß*-thalassemia genes has been achieved in the Chinese population, with coverage of other abnormal hemoglobin variant sites on *HBA1*, *HBA2* and *HBB* genes (Wu et al., 2022). This method can help with the accurate diagnosis and treatment of the disease and avoids the risk of missed detection. In this study, SMRT technology was used to detect four rare variants (large fragment deletions and gene recombination). Pedigree and phenotype analyses were also performed to explore the value of SMRT technology in the study of large fragment deletion and gene recombination of the globin gene cluster.

## Materials and methods

### Research subjects

#### Family A

The proband, registered in Liuzhou, Guangxi, China, was female, 40 years old. The genetic testing results were normal for common type α-thalassemia and 17 types of common Chinese *ß*-thalassemia. Her hemoglobin level was 99 g/L, which indicated microcytic hypochromic anemia, of unknown cause. The copy number variation of the α-globin gene cluster was assessed by MLPA technology and revealed an HBZP1 region-HBQ1-3 heterozygous deletion but the breakpoint was not clear. The family members of the proband were also analyzed. In June of the same year, one sporadic case was also found, registered in Liuzhou City, Guangxi. The patient was female, 30 years old and test results were normal for the common type of α-thalassemia and 17 types of common Chinese *ß*-thalassemia. Her hemoglobin level was 98 g/L, with small cell hypochromic anemia of unknown cause. The MLPA results showed an HBZP1 region-HBQ1-3 heterozygous deletion. We collected peripheral venous blood samples from all subjects in the study, using EDTA-K2 anticoagulation tubes. Signed informed consent was obtained from all subjects.

#### Family B

The proband, registered in Liuzhou, Guangxi, China, was female, 25 years old. The genetic testing results were normal for common type α-thalassemia and 17 common Chinese *ß*-thalassemia types. The hemoglobin level was 111 g/L, with microcytic hypochromic anemia of unknown cause. The HS-40 deletion was found by copy number analysis of the α-globin gene cluster but the deletion breakpoint was not clear. The family members of the proband were also analyzed.

#### Family C

The proband, registered in Laibin City, Guangxi, China, was male and 27 years old. He came to our hospital for a pregnancy test with his wife in 2020. The genetic testing results were normal for common type α-thalassemia and 17 types of common Chinese *ß*-thalassemia. The Hb F level was increased but the reason for this was not known. The *HBG1*-*HBG2* deletion was found by copy number analysis of the *ß*-globin gene cluster. The deletion breakpoint was not clear. His wife was a *ß*-thalassemia minor gene carrier. As there was a potential risk of having a hemoglobin intermediate *ß*-thalassemia fetus, the wife of the proband underwent prenatal diagnosis by amniocentesis at 22 weeks of gestation. The amniotic fluid of the fetus was tested by the conventional reverse dot blot technique for *ß*-globin gene mutation. The fetus was tested by MLPA for copy number variation of the *ß*-globin gene cluster. The family members of the proband were also analyzed. The wife chose to continue with the pregnancy and the child was followed up six months after birth.

#### Family D

The proband’s household registration was in Liuzhou City, Guangxi Province, China. She was female, 40 years old and came to our hospital for a pregnancy test in 2021. The routine genetic test found -α^3.7^/αα in the proband and her husband was --^SEA^/αα. As the parents were carriers of homotype α-thalassemia and at risk of giving birth to an intermediate α-thalassemia baby, we collected amniotic fluid and umbilical cord blood for prenatal diagnosis at 20 weeks and three days into pregnancy. Due to the abnormal results from the routine techniques, gene recombination was assessed using the α-globin gene cluster copy number variation test. The family members of the proband were also analyzed.

### Hematology and hemoglobin electrophoresis analysis

An automatic blood cell analyzer was used for routine blood analyses and high-performance liquid chromatography was used for hemoglobin analysis to detect hemoglobin F (Hb F), hemoglobin A2 (Hb A2), hemoglobin H (HbH) and other hemoglobin variants. Umbilical cord blood samples were analyzed for hemoglobin composition using a Capillarys 2 capillary electrophoresis analyzer (Sebia, France). The instrument was operated in accordance with the instruction manual and internal quality control was performed for each experiment.

### Genomic DNA extraction

The magnetic bead method was used to extract nucleic acids (LabAid820, Xiamen Zhishan Biotechnology, Xiamen, China). We then used a nucleic acid analyzer (ASP-2680; ACTGene, Piscataway, NJ, United States) to detect DNA concentration and purity. The A_260_/A_280_ was between 1.6 and 1.9 and the concentration was 20-30 ng/μL.

### α-thalassemia and β‐thalassemia genotyping analysis

Genomic DNA was extracted from peripheral blood using the thalassemia detection kit (Yishengtang, Shenzhen, China). The routine analysis for the four common α-thalassemia deletions [--^SEA^ (Southeast Asia), -α^3.7^ (rightward), -α^4.2^ (leftward) and --^THAI^ (Thailand)] was performed using Gap-PCR and three common non-deletion α-thalassemia mutations, Hb Constant spring (*HBA2*: c.427T > C), Hb Quong Sze (Hb QS, *HBA2*: c.377T > C) and Hb Westmead (*HBA2*: c.369G > C), were assessed. We also analyzed 17 known *ß*-thalassemia mutations; -28 (A > G) (*HBB*: c.-76A > G), -29 (A > G) (*HBB*: c.-79A > G), -30 (T > C) (*HBB*: c.-80T > C), -32 (C > A) (*HBB*: c.-82C > A), codons 14/15 (+G) (*HBB*: c.45_46insG), codon 17 (A > T) (*HBB*: c.52A > T), codon 26 (or Hb E) (G > A) (*HBB*: c.79G > A), codons 27/28 (+C) (*HBB*: c.84_85insC), codon 31 (–C) (*HBB*: c.94delC), codons 41/42 (–TTCT) (*HBB*: c.126_129delCTTT), codon 43 (G > T) (*HBB*: c.130G > T), codons 71/72 (+A) (*HBB*: c. 216_217insA), IVS-I-1 (G > T) (*HBB*: c.92+1G > T), IVSI-5 (G > C) (*HBB*: c.92+5G > C), IVS-II-654 (C > T) (*HBB*: c.316-197C > T), CAP+1(A > C) (*HBB*: c.-50A > C) and initiation codon (T > G) (*HBB*:c.2T > G). These mutations were detected by PCR and a reverse dot-blot assay (Shenzhen Yishengtang Biological Products Co., Ltd., Shenzhen, China). The MLPA detection method was performed, using the P102 and P140 probe kit (MRC-Honand. Netherlands), to analyze the copy number variation in deletional thalassemia genes. Capillary electrophoresis was performed on amplified products using a genetic 3500Dx analyzer (Applied Biosystems, Foster City, CA, United States).

### Third generation sequencing and data analysis

Experiments were conducted as described in a previous study. In brief, genomic DNA was amplified by PCR with primers that covered the majority of known structural variations, single nucleotide variations (SNVs), insertions and deletions (indels) in the *HBA1, HBA2*, *HBB, HBG* and *HS40* genes. The PCR products were ligated to barcoded adaptors by one-step end-repair and ligation reactions to construct pre-libraries. These were pooled together by equal mass and converted to a SMRT bell library by the Sequel Binding and Internal Ctrl Kit 3.0 (Pacific Biosciences). The SMRT bell library was then sequenced under the CCS mode on the Sequel II platform (Pacific Biosciences). After sequencing, subreads were converted to CCS reads, by CCS software (Pacific Biosciences), and debarcoded by lima in the Pbbioconda package (Pacific Biosciences). After alignment of the processed reads to genome build hg38, by pbmn2 (Pacific Biosciences), structural variations were identified in accordance with the HbVar, Ithanet and LOVD databases. The SNVs and indels were identified by FreeBayes1.3.4 (https://www.geneious.com/plugins/freebayes; Biomatters, Inc., San Diego, CA). Haplotype analysis was performed with WhatsHap (version 0.18) and pbampliconclustering software.

### The α-thalassemia and *ß*-thalassemia gene sequencing

Four primer pairs were designed and used to amplify and sequence the *HBA1* and *HBA2* genes: Sequences were as follows:

α1-F: 5′-TGG AGG GTG GAG ACG TCC TG-3'; α1-R: 5′-TCC ATC CTC TCC CGC CCC TGC CTT TTC-3'; α2-F: 5′-TGG AGG GTG GAG ACG TCT TG-3'; and α2-R: 5′-CCG TTG GCA CAT TCC GG-3'. *ß*-FP:5′-AAC TCC TAA GCC AGT GCC AG-3’; AvaII-β-FP:5′-TTG GGG ATC TGT CCA CTC CT-3’; AvaII-β RP:5′-CCA GCC TTA TCC CAA CCA TAA AAT AA-3’; *ß*-RP:5′-ATG CAC TGA CCT CCC ACA TTC CCT-3’. DNA sequencing was performed using the Sanger dideoxy termination method and the reference sequence was NM_000517. Amplification was performed using 50 ng of genomic DNA and 20 pmol of forward (F) and reverse (R) primers, on the C1000 thermal cycler (C1000 Thermal Cycler; Bio-Rad Laboratories, Hercules, CA, United States). The PCR products were sequenced on the ABI PRISM^®^ 3130 automated sequencer (Applied Biosystems).

### Data analysis

The results of the two methods were assessed and individuals with negative test results were classed as negative for thalassemia. Those with positive test results were diagnosed as thalassemia carriers. We calculated the coincidence rate for the results of the two methods. For the samples with inconsistent results between the two methods, verification by Sanger sequencing was performed (deletion and duplication mutations were verified by Q-PCR and MLPA technology) to determine which result was correct.

## Results

### Identification of a novel 27,311 bp deletion (--^27.3^/) that caused α^0^-thalassemia in family A and the sporadic case

**FIGURE 1:**
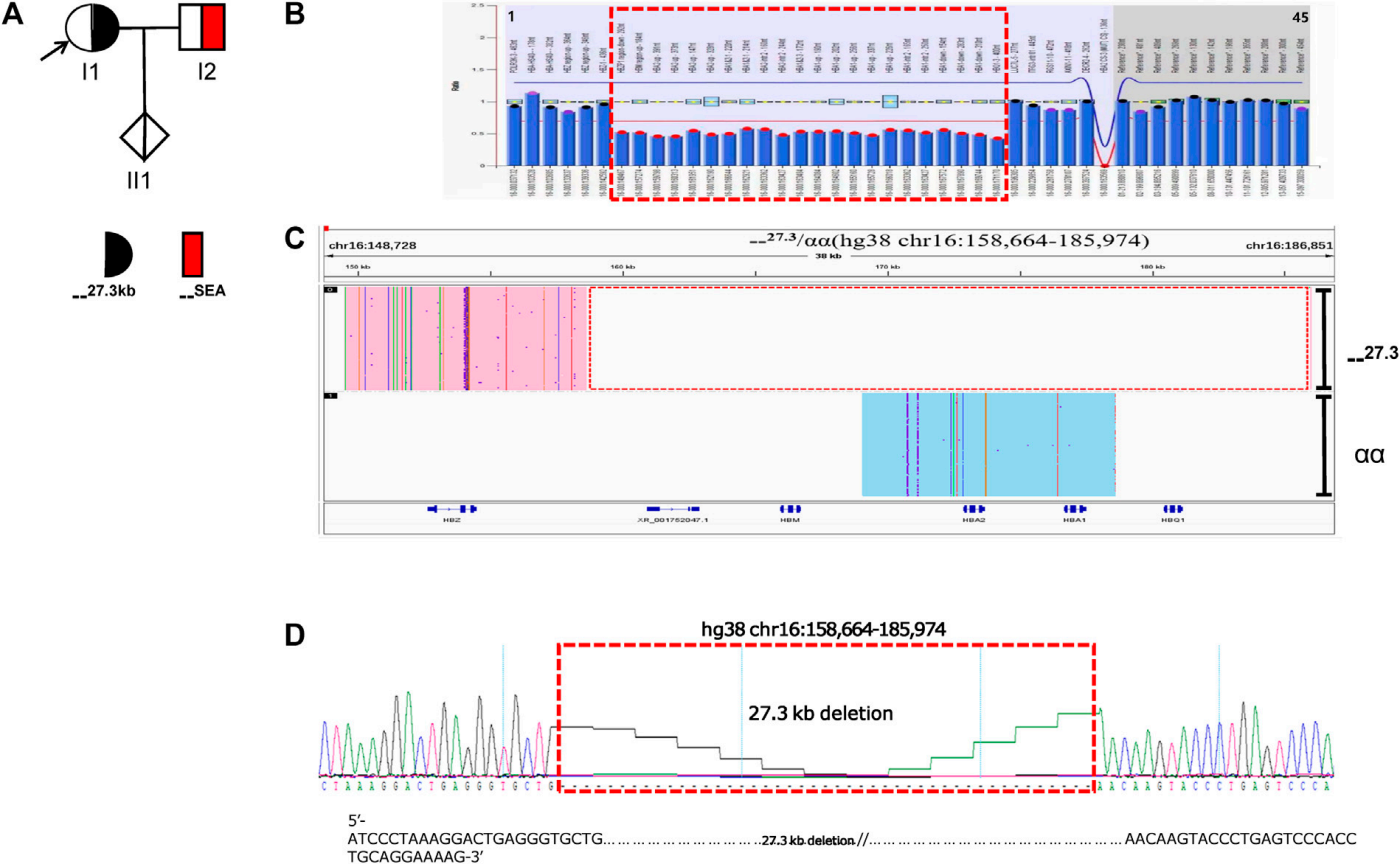
Family A.** (A):** Pedigree of the family A. The proband (I1) is heterozygous for the 27.3 kb deletion and her husband (I2) is heterozygous for the SEA deletion, while her fetus (II1) has normal α-genotype (no deletion).The arrow indicate the proband.** (B):** MLPA results for the proband (I1). We numbered the probes and labeled them prob-1 to prob-45 according to location (from left to right). Prob-35 to prob-45 were control probes with copy numbers corresponding to two copies of the genome, and when the other probes had a ratio of 1:1 to control probes, which were two normal copies. The proband (I1) had a ratio of 0.5 from prob-7 to prob-28, suggesting that the sample carried one copy of rare deletion.The deletion was highlighted in dashed red box. **(C):** The results of Long-read SMRT sequencing for the proband (I1). Integrative Genomics Viewer (IGV) plot of I1 showed one allele with αα in blue area and the other allele with 27.3/αα (hg38 chr16:158664-185974) novel deletion in pink area. The deletion was highlighted in dashed red box.Chr, chromosome. **(D): **Sanger sequencing for the proband (I1).Sequencing result of 27.3 kb deletion (primer sequences F:AAAATCAACAAGGACATTAGGC, R:CGTGTCCGTATTCCTCCC) in α-globin gene of the I1 sample. The deletion was highlighted in dashed red box.

The proband of family A had microcytic hypochromic anemia but no abnormality was found in routine genetic testing and their ferritin result was normal. The pedigree chart showed in Figure 1A. Due to the discordance between the thalassemia genetic testing and the clinical hematologic phenotype, other types of mutations were considered. No mutations were found in the *HBA* and *HBB* genes by Sanger sequencing. An MLPA analysis was performed to identify the presence of deletions in the α-globin gene cluster. This showed that the proband carried a large deletion that contained the *HBZP1*, *HBM*, *HBA1*, *HBA2* and *HBQ1* genes (Figure 1B). Due to the limited number of MLPA probes, the deletion breakpoint could not be identified and, subsequently, SMRT was performed to pinpoint the deletion extent and breakpoint. SMRT confirmed the MLPA results and indicated the presence of a heterozygous deletion in the mother, which had been inherited by the proband (Figure 1C). Bioinformatics analysis successfully positioned the breakpoints at chromosome 16:158664-185974 (GRCh38) and predicted the deletion to be approximately 27311 bp in size and Sanger sequencing through designed primers were consistent with the SMRT sequencing (Figure 1D). The proband (I1) had a heterozygous 27.3 kb deletion in the α-globin gene cluster (--^27.3^/αα (hg38 chr16:158664-185974), which resulted in the expression of only two functional α-globin genes, reduced mean corpuscular volume (MCV) and reduced mean corpuscular hemoglobin (MCH). The proband had α0-thalassemia characteristics and a hematological phenotype that was similar to --^SEA^ (NC_000016.10: g.165401_184701del) carriers. Similarly, we found a sporadic case of the α-globin gene cluster 27,311 bp deletion, --^27.3^/αα, that also had α^0^-thalassemia features, reduced *MCV* and reduced *MCH* (Table 1).

**TABLE 1:** Hematological data and α/β-globin genotype of case.

Case		Sex-age		Hb (g/L)		RBC (10*12/L)		MCV(fL)		MCH(pg)		MCHC(g/L)		Hb A2 (%)		Hb A (%)		Hb F (%)	Hb Bart's(%)	α Genotype (Gap-PCR+RDB)	β Genotype (RDB)	α or β Genotype (MLPA)	α or β Genotype (SMRT)
Family A																							
I-1	Proband(mother)	M-40		99		4.46		66.5		22.1		332		2.3		97.7		–	–	αα/αα	β^N^/β^N^	Normal	hg38 chr16:158664-185974
I-2	Father	F-39		152		7.45		69		21		307		2.3		97.7		–	–	--^SEA^/αα	β^N^/β^N^	--SEA/αα	^--SEA^/αα
II-1	Fetus	25W+4D		–		–		–		–		–		–		–		–	–	αα/αα	β^N^/β^N^	Normal	αα/αα
Sporadic case																							
I-1	Sporadic case	M-38		98		4.28		72.2		22.8		316		2.6		97.4		0	–	αα/αα	β^N^/β^N^	*HBA1、HBA2 deletion*	hg38 chr16:158664-185974
Family B																							
I-1	Proband(mother)	M-25		111		4.69		71.5		23.6		330		2.5		87.1		0.4	–	αα/αα	β^N^/β^N^	*HS-40 deletion*	hg38 chr16:100600-116678
I-2	Father	F-25		136		6.82		62.9		19.9		316		5.3		83.8		1.5	–	αα/αα	*HBB:c.126_129delCTTT*	αα/αα and βN/βN	*HBB:c.126_129delCTTT*
II-2	Proband	F-3		119		5.76		65.1		20.7		317		2.1		93.9		4	–	αα/αα	*HBB:c.126_129delCTTT*	*HS-40 deletion*	hg38 chr16:100600-116678; *HBB:c.126_129delCTTT*
Family C																							
I-1	Mother	M-51		125		4.06		93.2		30.8		331		2.7		97.3		0	–	αα/αα	β^N^/β^N^	*HBG1-HBG2 deletion*	hg38 chr11:5249345-5254268
I-2	Father	F-55		129		5.73		70.3		22.5		320		4.8		89.5		5.7	–	αα/αα	*HBB:c.-78A>G*	Normal	*HBB:c.-78A>G*
II-1	Sister	M-35		118		5.16		74.9		22.9		306		4.1		91.6		4.3	–	αα/αα	*HBB:c.-78A>G*	Normal	*HBB:c.-78A>G*
II-2	Proband	F-27		139		4.45		93		31.3		329		2.1		93.9		4	–	αα/αα	β^N^/β^N^	*HBG1-HBG2 deletion*	hg38 chr11:5249345-5254268
II-3	Wife	M-21		110		5.77		58.8		19		325		5.6		93		1.4	–	αα/αα	*HBB:c.126_129delCTTT*	Normal	*HBB:c.126_129delCTTT*
II-4	Younger brother	M-26		129		5.55		73.5		23.2		316		5.4		91.8		2.8	–	αα/αα	*HBB:c.-78A>G*	Normal	*HBB:c.126_129delCTTT*
III-1	Fetus	22W	–		–		–		–		–		–		–		–		–	αα/αα	*HBB:c.126_129delCTTT*	*HBG1-HBG2 deletion*	*hg38 chr11:5249345-5254268;HBB:c.126_129delCTTT*
		F-3D	165		4.75		103		34.8		338		0		15		85						
		F-6M	106		5.53		60.6		19.2		317		–		–		–						
Family D																							
I1	Mother	M-70		121		5.75		65.4		21.1		322		5.2		94.4		0.4	-	αα/αα	*HBB:c.-78A>G*	Normal	*HBB:c.-78A>G*
II1	Sister	M-54		132		4.59		86.8		28.7		331		2.5		96.5		1	-	-α^3.7^/αα	β^N^/β^N^	-	-ɑ^3.7^ɑ1ɑ2/ɑɑ
II4	Brother	F-43		129		5.75		69.9		22.4		320		5.5		94.5		0	-	-α^3.7^/αα	*HBB:c.-78A>G*	-	-ɑ^3.7^ɑ1ɑ2/ɑɑ; HBB:c.-78A>G
II5	Proband	M-40		117		3.89		87.9		30		341		2.4		97.6		0	-	-α^3.7^/αα	β^N^/β^N^	-	-ɑ^3.7^ɑ1ɑ2/ɑɑ
II6	Husband	F-38		141		3.92		75		23.2		336		2.4		96.9		0.7	-	--SEA/αα	β^N^/β^N^	--^SEA^/αα	--^SEA^/αα
III1	Son	F-8		113		5.33		64.5		21.2		328		2.2		97		0.8	-	--SEA/αα	β^N^/β^N^	--^SEA^/αα	-^-SEA^/αα
III2	Fetus	20W+3D		–		–		–		–		–		–		7.3		79.2	13.5	-ɑ^3.7^/ɑ1ɑ2/--^SEA^	β^N^/β^N^	-ɑ^3.7^/ɑ1ɑ2/^--SEA^	-ɑ^3.7^/ɑ1ɑ2/--SEA

Abbreviations: RBC, red blood cell count Hb, hemoglobin. MCV, mean corpuscular volume. MCH, mean corpuscular Hb.βN, 17 β-thalassemia genotypes commonly found in the Chinese population was not found.--SEA, Southeast Asian deletion, NG_000006.1:g.26264_45564del19301.-α3.7, 3.7-kb deletion of HBA,NG_000006.1:g.34164_37967del3804.

### Identification of a novel 16.1 kb deletion of HS-40 that caused thalassemia in family B

**FIGURE 2:**
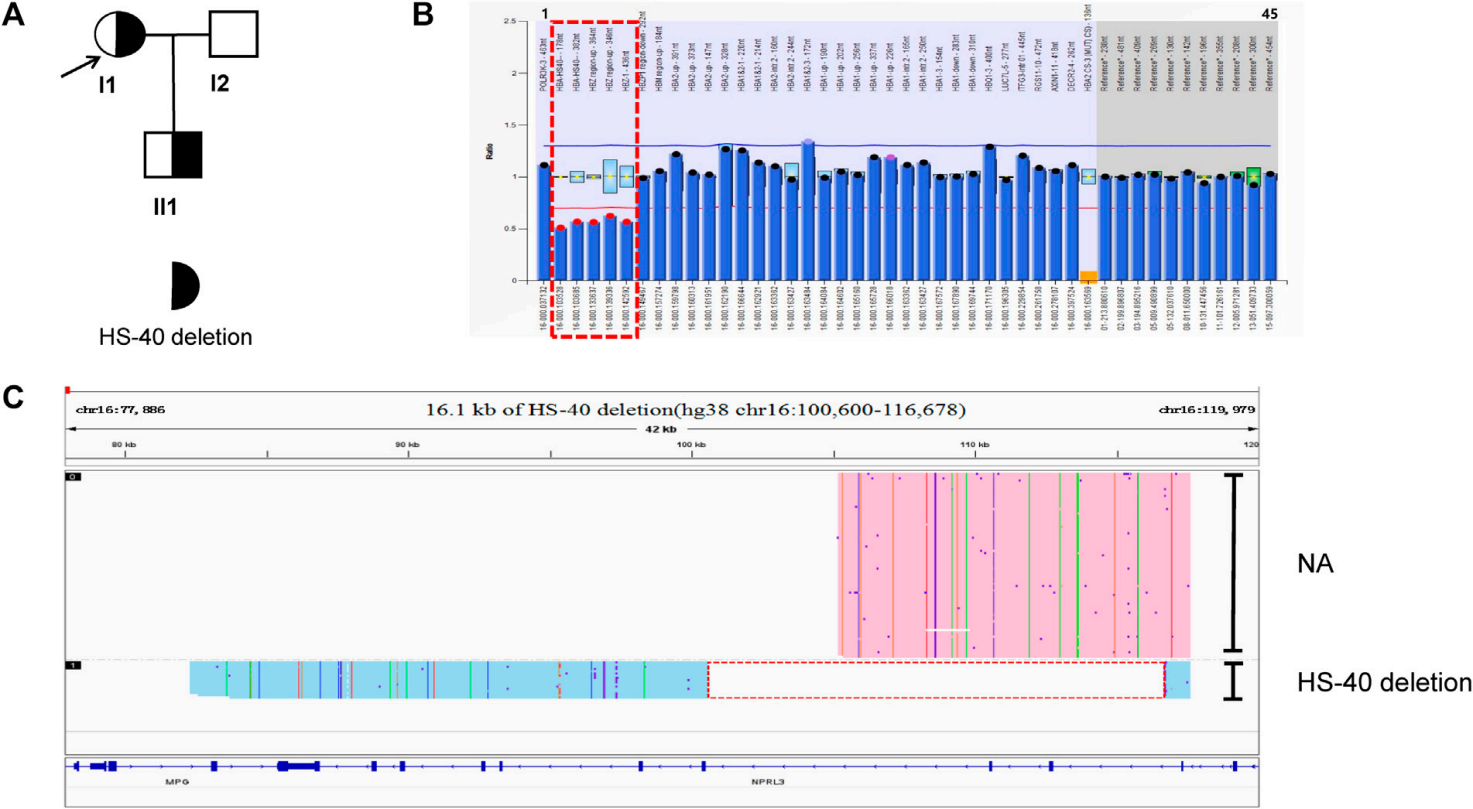
Family B. **(A):** Pedigree of the family B. The proband (I1) and her son (II1) are heterozygous for the HS-40 deletion, while her husband (I2) has normal α-genotype (no deletion). The arrow indicate the proband. **(B):** MLPA results for the proband (I1). We numbered the probes and labeled them prob-1 to prob-45 according to location (from left to right). Prob-35 to prob-45 were control probes with copy numbers corresponding to two copies of the genome, and when the other probes had a ratio of 1:1 to control probes, which were two normal copies. The proband (I1) had a ratio of 0.5 from prob-2 to prob-6 suggesting that the sample carried one copy of rare thalassemia deletion. The deletion was highlighted in dashed red box. **(C)**: The results of Long-read SMRT sequencing for the proband (I1). IGV plot of sample I1 showed a 16.1 kb deletion in the HS-40 region of the α-globin gene cluster (hg38 chr16:100600-116678) in blue area. The deletion was highlighted in dashed red box. Chr, chromosome. NA indicates that no variant was detected.

The family B proband (I1) and her son (II1) had microcytic hypochromic anemia. Routine genetic testing revealed no abnormalities, which suggested the possibility of an unknown mutation, The pedigree chart showed in Figure 2A. No mutations were found in the *HBA* and *HBB* genes by Sanger sequencing. The MLPA analysis revealed that the proband was heterozygous for a deletion in the upstream regulatory element, HS-40, of the *HBA* gene. The son (II1) also had a heterozygous deletion that involved HS-40, with a missing breakpoint (Figure 2B). Subsequently, SMRT was used to confirm MLPA results and indicated the presence of a heterozygous deletion in the mother, which had been inherited by the proband. Bioinformatics analysis successfully positioned the breakpoints at chromosome 16:100600-116678 (GRCh38) and predicted the deletion to be approximately 16.1 kb in size (Figure 2C). The hematological features of the mother with the 16,079 bp deletion in the HS-40 region (hg38 chr16:100600-116678) were as follows: Hb 111 g/L, MCV 71.5 fL, MCH 23.6 pg, 0.4% HbF and 2.5% HbA2) (Table 1). Both cases with this deletion presented with microcytic hypochromic anemia.

### Identification of a novel 4.9 kb deletion that caused thalassemia in family C

**FIGURE 3:**
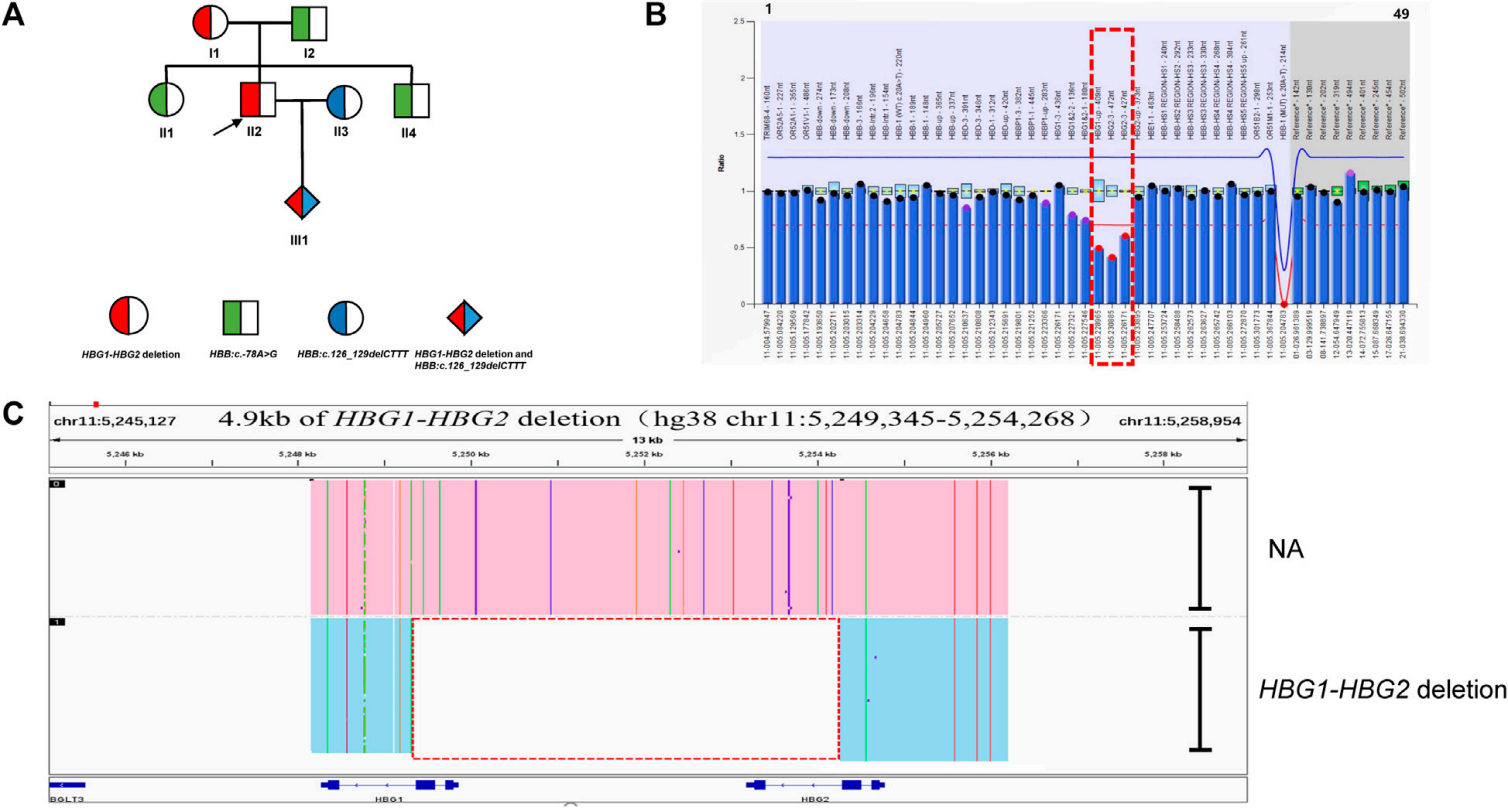
Family C. **(A):** Pedigree of the family C. The proband (II2) and his mother (I1) are heterozygous for the HBG1-HBG2 deletion, while his father (I2) and sister (II1) and brother (II3) are heterozygous for the HBB:c.-78A>G variant. The arrow indicate the proband. **(B):** MLPA results for the proband (II2). We numbered the probes and labeled them prob-1 to prob-49 according to location (from left to right). Prob-41 to prob-49 were control probes with copy numbers corresponding to two copies of the genome, and when the other probes had a ratio of 1:1 to control probes, which were two normal copies. The proband (II2) had a ratio of 0.5 from prob-26 to prob-28 suggesting that the sample carried one copy of rare deletion in HBG1-HBG2 area. The deletion was highlighted in dashed red box. **(C):** The results of Long-read SMRT sequencing for the proband (II2). IGV plot of sample II2 showed a 4.9 kb HBG1-HBG2 deletion in the β-globin gene cluster (hg38 chr11:5249345-5254268) in blue area. The deletion was highlighted in dashed red box. Chr, chromosome. NA indicates that no variant was detected.

In family C (Figure 3A), routine blood parameters of the proband were found to be normal but the HbF levels were increased. A SMRT analysis confirmed MLPA results (Figure 3B) and indicated the presence of a heterozygous deletion in the proband, which was inherited by the proband. Subsequently, third generation sequencing was performed to pinpoint the deletion extent and breakpoints. Bioinformatics analysis successfully positioned the breakpoints at chromosome 11:5249345-5254268 (GRCh38) and predicted the deletion to be approximately 4.9 kb in size (Figure 3C). The proband (II-2) and his mother (I-1) were carriers of the 4.9 kb deletion (hg38 chr11:5249345-5254268) in the *ß*-globin gene cluster, HBG1-HBG2. The father (I-2), sister (II-1) and younger brother (II-4) carried *HBB*:c.-78A > G, whilst his wife (II-3) carried *HBB*:c.126_129delCTTT. The fetus (III-1) had *HBB*:c.126_129delCTTT, with the *HBG1*-*HBG2* 4.9 kb deletion. The MCV and MCH levels in the proband were normal. The HbA2 content was decreased, whilst the HbF content was increased. The mother’s hematological phenotype was normal, whilst the father (I-2), sister (II-1) and younger brother showed mild hypochromic anemia, increased HbA2 content and slightly increased HbF content in the father (I-2) and sister (II-1). The wife has microcytic hypochromic anemia and her HbA2 level was increased (Table 1). The son had microcytic hypochromic anemia after birth and his hemoglobin level reached mild anemia.

### A case and pedigree analysis of the allelic -α^3.7^α1α2 gene rearrangement

**FIGURE 4:**
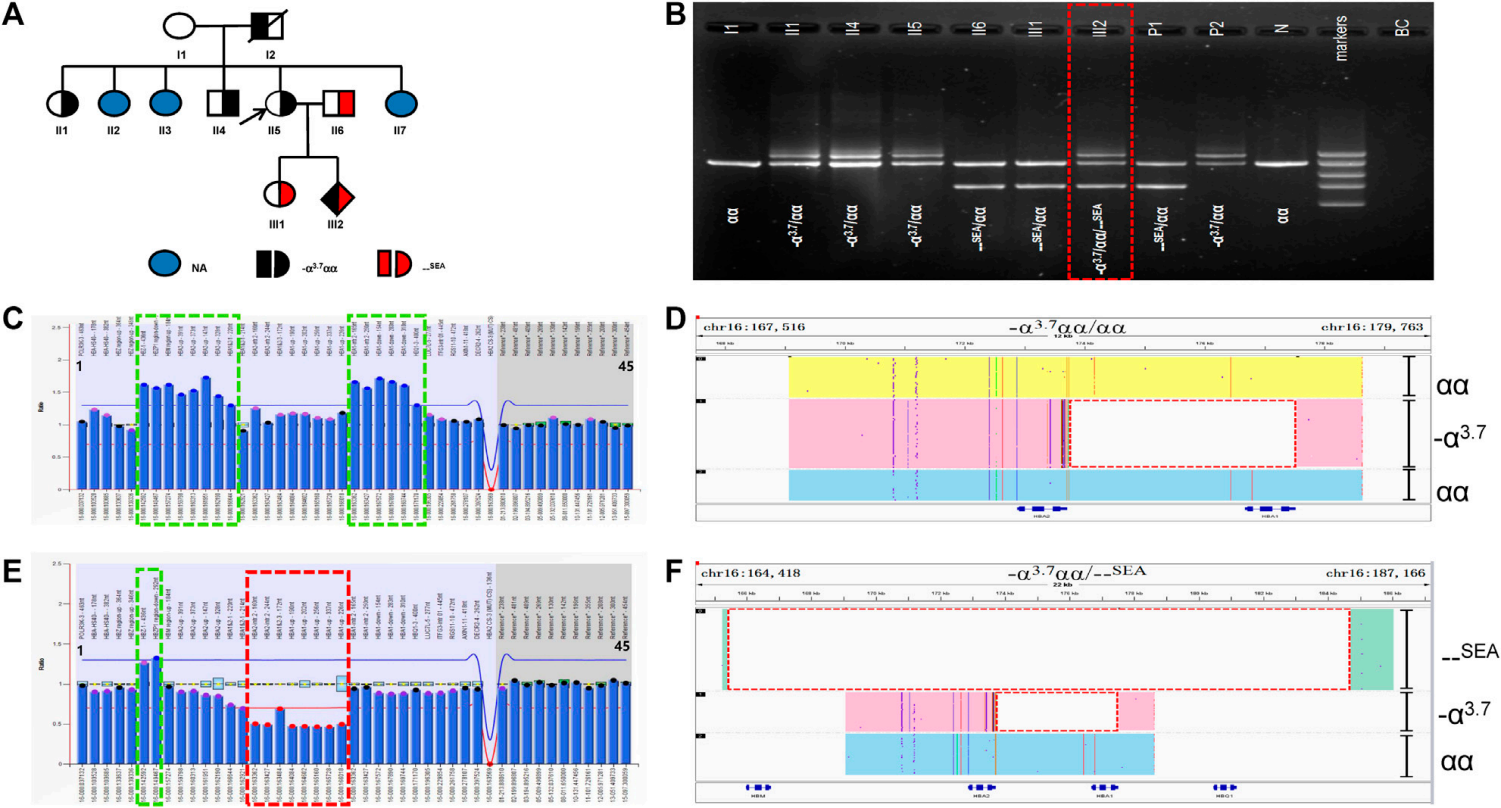
Family D. **(A):** Pedigree of the family D. The proband (II5) and her father (I2) and her sister (II1) and her brother (II4) are heterozygous for the –α3.7 deletion, while his husband (II6) and her daughter (III1) are heterozygous for the SEA deletion. Her fetus (III2) has three alleles (–α3.7, αα, SEA). The arrow indicate the proband. NA indicates that no variant was detected. **(B):** Gap-polymerase chain reaction (Gap-PCR) and agarose gel electrophoresis showing the expected amplicons for different genotypes. Three electrophoresis bands of 1.2 kb, 1.7 kb and 2.0 kb (SEA, αα, –α3.7) were detected in the fetus (III2) .The deletion was highlighted in dashed red box. **(C,E):** MLPA results for the proband (II5) and her fetus (III2). We numbered the probes and labeled them prob-1 to prob-45 according to location (from left to right). Prob-35 to prob-45 were control probes with copy numbers corresponding to two copies of the genome, and when the other probes had a ratio of 1:1 to control probes, which were two normal copies. The proband (II5) had a ratio of 1.2-1.4 from prob-6 to prob-13 and prob-23 to prob-28 suggesting that the sample carried three copies of duplication. While Her fetus (III2) had a ratio of 1.2-1.4 from prob-6 to prob-7 and a ratio of 0.5 from prob-15 to prob-22 suggesting that the sample carried one copy of deletion and three copies of duplication. **(D,F):** The results of Long-read SMRT sequencing for the proband (II2) and her fetus (III2). IGV plot of sample II2 showed–α3.7 deletion in pink area, one αα allele in yellow area, one αα allele in blue area. IGV plot of sample IIi2 showed –α3.7 deletion in pink area, SEA deletion in green area, one αα allele in blue area. The deletion was highlighted in dashed red box.

In family D (Figure 4A), the routine blood parameters of the proband were normal. Routine genetic testing found that the proband was -α^3.7^/αα and her husband was --^SEA^/αα. Both the proband and her husband were carriers of α-thalassemia intermedia. When there is a risk of α-thalassemia, prenatal diagnosis in pregnant women is performed using amniotic fluid and umbilical cord blood at 20 weeks and 3 days into pregnancy. Prenatal diagnosis by fetal Gap-PCR revealed abnormal bands (Figure 4B). The RDB results were abnormal and the MLPA analysis revealed an abnormal α-globin copy number (Figure 4C). The SMRT results showed a rearrangement of the -α^3.7^α1α2 gene on one allele but it was not possible to determine whether -α^3.7^ was spliced in the upstream or downstream region of α1α2 (Figure 4D). Analyses of the family and the phenotype of the rare variant were also carried out (Table 1). One -α^3.7^α1α2 gene rearrangement was detected in four cases (II1, II4, II5 and III2). II1 and II5 carried only gene rearrangements, and hematological parameters were normal. The II4 gene rearrangement was consistent with *HBB*:c.-78A > G and the individual had microcytic hypochromic anemia, an HbA2 content of 5.5% and an HbF content of 0%. The III2 individual was a fetus and prenatal diagnosis indicated a gene rearrangement that was consistent with --^SEA^/αα (Figures 4E,F). The Hb Bart’s content of the umbilical cord blood was 13.5%, as shown by electrophoresis, and III2 had the Hb H disease phenotype.

## Discussion

In recent years, the application of SMRT sequencing technology has been popular for the genetic detection of thalassemia (Xu et al., 2020; Liang et al., 2021; Jiang et al., 2022; Luo et al., 2022; Wu et al., 2022). The technology has a wide detection range, high accuracy and is able to detect structural variations in *HBB* and *HBA1/HBA2* gene mutations. It is particularly useful for the discovery of new mutations in *HBB* and *HBA* genes and for the differential diagnosis of trans or cis variants, such as -α^3.7^, -α^4.2^, ααα^anti3.7^, ααα^anti4.2^ and HKαα. Using the powerful PacBio detection system, a high throughput level of simultaneous detection of 384 samples can be achieved, which makes the cost of a test less than $20 (Wu et al., 2022). In this study, the 27,311 bp deletion (--^27.3^) and 16,079 bp deletion in the HS-40 region of the α-globin gene cluster were reported and located for the first time by SMRT sequencing technology. One allele of the α-globin gene cluster α^3.7^α1α2 rearrangement and a *ß*-globin gene cluster *HBG1*-*HBG2* 4,924 bp deletion were also reported. Phenotype and pedigree analyses were performed for the four rare variants (Luo et al., 2022). The limitations of conventional techniques mean that they cannot be used to accurately diagnose these four rare variants. Techniques such as Gap-PCR and PCR-RDB can only detect common types of thalassemia, which include four deletion α-thalassemias, three non-deletion α-thalassemias and 17 *HBB* gene mutations. Using MLPA, globin copy number variations, such as duplications and deletions, can be identified on the α-globin and *ß*-globin gene clusters but the lack of detection probes means that this technology cannot accurately locate deletion breakpoints and structures. Sanger sequencing technology is suitable for SNVs, indels and point mutations but it is very difficult to detect large fragment deletions or duplications using this method. It is possible to combine Gap-PCR and Sanger sequencing technology to design primers near the deletion breakpoint, in order to identify and locate unknown mutations. However, this is very time consuming, with a high mutation rate of primers, low detection throughput and issues with the existence of pseudogenes and highly homologous genes (Krier et al., 2016). The α-globin gene cluster 27,311 bp deletion, -^−27.3^/αα, results in the expression of only two functional α-globin genes. Individuals with heterozygous deletions (e.g., I2 and the sporadic case) have the clinical hematological features of α^0^-thalassemia, with reduced MCV and MCH and a deletion size and region similar to the --^SEA^ deletion. The carrier phenotypes are also very similar. The clinical case with the 16,079 bp deletion in the HS-40 region had microcytic hypochromic anemia. The HS-40 region contains an erythroid-specific DNase I high-sensitivity site that is 40 kb upstream of the α-globin gene cluster. It is an important regulatory site that affects the expression of α-globin and its deletion affects downstream α-globin expression (Harteveld et al., 2002; Espéret et al., 2000). In this case, the deletion on one chromosome reduced the expression of α1 and α2 globin. The patient presented with microcytic hypochromic anemia and mild anemia, which was similar to the hematological phenotype of patients with pure --^SEA^/αα. This indicated that deletion of this locus leads to a decrease in the expression of α-globin, as in patients with --^SEA^/αα. Our previous studies were consistent with this result. The deletion of the HS-40 locus, combined with the Southeast Asian type deletion α-thalassemia, will manifest as papilledema fetuses (Luo et al., 2020).

On the *ß*-globin gene cluster, one gene is expressed in the fetal period (ε), two genes are expressed in infancy (Gγ and Aγ), two genes are expressed in the adult period (β and δ) and there is one pseudogene (ψβ). The order of the genes on the gene cluster is 5′-ε-Gγ-Aγ-ψβ-δ-β-3'. Upstream of the ε gene, there is a locus control region (LCR), which is crucial for the expression of all genes on the *ß*-globin gene cluster. The Gγ and Aγ globin genes (*HBG1* and *HBG2*) are commonly expressed in the fetal liver, spleen and bone marrow. The predominant fetal hemoglobin (HbF, α2/γ2) consists of two *γ* chains and two α chains, which are usually replaced by adult hemoglobin (HbA, α2/β2) at birth. In some *ß*-thalassemias and related diseases the production of *γ* chains persists into adulthood and can be phenotyped as persistent fetal hemoglobinemia. The two gamma chains of the Gγ and Aγ globin proteins differ at site 136, where glycine is found in the Gγ protein (*HBG2*) and alanine is found in the Aγ protein (*HBG1*). The Gγ version is predominant at birth (Kiefer et al., 2011; Huang et al., 2017). This study reported a case of a 4,924 bp deletion (hg38 chr11:5249345-5254268) in the *ß*-globin gene cluster, which covers the *HBG1*-*HBG2* region. A study has reported that this Aγ-IVS2 regulatory region may affect HbF regulation in adults (Donovan-Peluso et al., 1987). The *HBG2*-*HBG1* fusions cause *?*-thalassemia and have been described in the literature (Shimizu et al., 1986; Jiang et al., 2020). However, in the cases that we reported, the blood cell parameters were normal and the HbF content was normal or increased, which is inconsistent with the above literature. It may be that the function of the *HBG1*-*HBG2* gene is turned off in adults and there are differences in the impact on the hematological phenotype (Lee et al., 2010; Chaouch et al., 2020). The wife carried *HBB*: c.126_129delCTTT and showed microcytic hypochromic anemia and increased HbA2 content. Three days after the birth of the son of the proband, heel blood was collected for hemoglobin electrophoresis. No abnormality was found in HbA or HbF levels. Analysis of his genetic tests showed that he had inherited his mother’s *HBB*: c.126_129delCTTT mutation and his father’s *HBG1*-*HBG2* 4,924 bp deletion. Retrospective analysis of his blood for six months showed that he had mild microcytic hypochromic anemia. This showed that *HBB*: c.126_129delCTTT with the *HBG1*-*HBG2* 4,924 bp deletion did not reach intermediate anemia. The hematological phenotype of the co-inherited cases of the two mutations was similar to the phenotype of the *ß*-thalassemia minor genotype and this is the first report of the effect of this deletion position on the phenotype of *ß*-thalassemia. The effect of the *HBG1*-*HBG2* 4,924 bp deletion on HbA2 and HbF varied among individuals, which increases the amount of data available for research.

α-globin polyploidy is ubiquitous in nature. The α-globin gene cluster has three homologous X-, Y- and Z-sequences and misplacement or unequal exchange between them, during meiosis, leads to the generation of triploids of the α-globingene. An exchange between the homologous Z2 and Z1 boxes (cross to the right) would result in a -α^3.7^ monoallelic deletion and a triple ααα^anti−3.7^ allele. If there is a crossover between the X2 and X1 boxes (crossing to the left), there will be a -α^4.2^ monoallelic deletion and a triple ααα^anti−4.2^ allele (Wang et al., 2003). Since the triple alleles still have intact α1 and α2 genes, ααα^anti−4.2^ and ααα^anti−3.7^ will be misdiagnosed as αα, based on conventional Gap-PCR detection. Studies have shown that approximately 2% of α-globin triple alleles are present in individuals with moderate to severe *ß*-thalassemia (Sollaino et al., 2009; Shooter et al., 2015). The coexistence of α-globin polyploidy and *ß*-thalassemia can aggravate the β/α imbalance and results in intermediate *ß*-thalassemia. In our case, through SMRT sequencing technology, it was clear that one chromosome of the mother was normal, whilst the other had complete α2α1 and -α^3.7^, to give a total of five copies of α-globin. Conventional Gap-PCR testing would misdiagnose this individual as -α^3.7^/αα. The MLPA technique can determine the existence of the five copies of α and the -α^3.7^ deletion but cannot determine the distribution. In the II1, II4, II5 and III2 cases, complete α2α1 and -α^3.7^ were detected on one chromosome and, in II1 and II5, one chromosome was normal, whilst the other had complete α2α1 and -α^3.7^, to give a total of five copies of α-globin. The hematological phenotype was normal. It is particularly noteworthy that the II4 case had a total of five copies of the α gene that were co-inherited with *HBB*: c.-78A > G, which aggravated the β/α imbalance. Previous literature has reported that this would generally cause intermediate *ß*-thalassemia (Sollaino et al., 2009; Shooter et al., 2015). However, in this case, the individual showed only mild *ß*-thalassemia. One chromosome of the III2 fetus was --^SEA^, whilst the other was inherited from the mother’s three recombined α-globin genes. Generally speaking, the presence of three normal copies of α-globin would produce a quiescent or mild α-thalassemia phenotype but, in fact, umbilical cord blood electrophoresis showed that the Hb Bart’s content reached 13.5%, which can be diagnosed as Hb H disease. The SMRT sequencing technology confirms that one chromosome of the mother was normal, whilst the other had complete α2α1 and -α^3.7^. There was a total of five copies of the α-globin gene, but it is still not clear whether -α^3.7^ was spliced in the upstream or downstream region of α2α1. Through analysis of the phenotypic differences between the above two cases, we speculated that -α^3.7^ recombination may have occurred at the 5′ end, upstream of α2α1. Since the variant occurred in the upstream region, it may affect the expression of downstream α2α1, which requires further research.

## Conclusion

In conclusion, this study demonstrated that SMRT sequencing technology is suitable for the detection of unknown deletions and complex structural variants in thalassemia cases. Compared with traditional methods, this technology is more effective and accurate. A single test can identify the breakpoints and structural variations of unknown deletions, within a short experimental period and at low cost. Due to its high technical requirements, SMRT has not yet been popularized in clinical practice. We believe that this technique shows great promise for the detection of unknown deletions and complex structural variants in thalassemia cases.

## Data Availability

The datasets for this article are not publicly available due to concerns regarding participant/patient anonymity. Requests to access the datasets should be directed to the corresponding authors.
